# Effects of progressive functional ankle exercises in spastic cerebral palsy, plantarflexors versus dorsiflexors: a randomized trial

**DOI:** 10.55730/1300-0144.5682

**Published:** 2023-05-31

**Authors:** Melek VOLKAN YAZICI, Gamze ÇOBANOĞLU, Gökhan YAZICI, Bülent ELBASAN

**Affiliations:** 1 Department of Physical Therapy and Rehabilitation, Faculty of Health Sciences, Yüksek İhtisas University, Ankara, Turkiye; 2 Department of Physical Therapy and Rehabilitation, Faculty of Health Sciences, Gazi University, Ankara, Turkiye

**Keywords:** Cerebral palsy, exercise, gastrocnemius muscle, anterior tibial muscle, posture balance, surface electromyography

## Abstract

**Background/aim:**

Children with cerebral palsy (CP), even those who have very mild impairment, have lower muscle strength than their typically developing peers. The ankle dorsiflexors (DFs) and plantarflexors (PFs) of children with CP are especially weak. Weakness in the ankle muscles causes problems in functional skills, mobility, and balance in spastic CP (SCP). The aim of this study was to investigate the effects of progressive functional exercises (PFEs) on the DF, PF, or dorsi-plantar flexor (DPF) muscles in children with SCP, specifically, the functional mobility, balance, and maximum voluntary contraction (MVC), and compare the effects of strengthening these muscles individually or combined.

**Materials and methods:**

This randomized trial was conducted between December 1st, 2018, and May 15th, 2019, at Gazi University, Department of Physiotherapy and Rehabilitation. Randomly assigned into groups were 27 independently ambulant patients with unilateral/bilateral SCP, where PFEs were applied to the DF, PF, or DPF muscles. Muscle tone, balance, and functional mobility were assessed. The MVC was evaluated by surface electromyography. PFEs were performed 4 times a week, for 6 weeks.

**Results:**

The spasticity of the PF muscles decreased in all of the groups. PFE of the DF muscles led to an increase in ankle joint range of motion (ROM) and improved functional mobility (p < 0.05). PFE of the PF muscles resulted in improvements in balance and functional mobility (p < 0.05). PFE of the DPF muscles brought about improvements in balance but not in functional mobility (p < 0.05). No significant difference in the MVC was observed in any of the groups (p > 0.05).

**Conclusion:**

Gains are obtained according to the function of a muscle group. By training the DF muscles, it is possible to improve function and ROM. Furthermore, training the PF muscles led to improvements in balance and functional mobility, indicating that it is possible to bring about positive changes in spastic muscles. This study showed that muscle groups must be exercised according to the intended goal.

## 1. Introduction

Spastic cerebral palsy (SCP) is the most common type of CP, which leads to motor deficits and disorders in the development of movement and posture. Motor deficits of SCP include negative phenomena such as weakness, fatigue, incoordination, and positive phenomena such as spasticity, clonus, rigidity, and spasms [1]. As a result of these deficits, children with SCP are likely to have developmental delays in gross motor function, standing, walking, and balance when compared to normally developing children [2].

Weakness is a prevailing impairment in individuals with CP and is caused by impaired neural ability and altered intrinsic capacity of the muscles [3]. Studies investigating the relationship between spasticity and strength in SCP have demonstrated that muscle weakness has a direct effect on function and leads to more limitations in function than spasticity [4–6]. Muscle weakness in SCP was reported to be associated with deficiencies in walking, running and a decrease in functional scales, consequently, weakness causes a restriction in participation in daily life activities [5, 7, 8]. Children with CP, even those who have very mild impairment, are weaker than their typically developing peers [9, 10]. For example, compared to the strength of the muscles in children with typical development, the muscle strengths of the ankle dorsiflexors (DFs) and plantarflexors (PFs) of children with CP were about 50% and 35% compared to their typically developing peers, respectively [11]. The DFs were found to be far weaker compared to the PFs [3]. Previously, the muscles of children with CP were thought to be incapable of adapting; however, it is now known that muscles in CP are indeed plastic [12]. The adaptations and improvements that can occur following strength or power training in the muscles of children with CP has been shown; however, the effect of progressive functional exercises (PFEs) is still unclear [7, 13, 14].

The foot and ankle joints are affected in almost all individuals with CP [10]. A significant decrease was demonstrated in tibialis anterior activation in children with CP when compared to their typically developing peers [15]. The PFs of the foot, which are the gastrocnemius and soleus muscles, resist and control the forward rotation of the tibia over the foot during the mid and terminal stance phases of gait [16]. The eccentric, antigravity activity of the soleus muscle is of primary importance due to modulating the site of application of the ground reaction force on the foot during weight bearing, thus allowing a biomechanical advantage in ambulation [17, 18].

It has been demonstrated that in patients with CP, exercises targeting the knee and hip muscles have beneficial effects on gait and function [19–22]. However, studies demonstrating the effects of exercises applied on ankle muscles on functional mobility are insufficient, and there are no studies examining the effect of PFE of these muscles on balance or muscle activation [23, 24]. PFE is a type of exercise with an overall goal of functional independence, which helps patients develop skills to perform activities of daily life easier [25]. PFEs consist of many daily activities involving the lower limbs, such as sit-to-stand and stair climbing [26]. It was reported that when exercises involve practicing functional closed-kinetic-chain exercises, gains in strength may lead to greater improvements in functional motor performance [26, 27]. Therefore, the primary aim of this study was to investigate the effects of PFE on muscle tone, balance, and functional mobility in patients with SCP. The second aim was to investigate the changes in the maximum voluntary contractions (MVC) of the lower extremity muscles following 6-weeks of PFE using surface electromyography (sEMG).

The primary hypothesis herein was that balance and functional mobility would improve following PFEs in patients with SCP.

## 2. Materials and methods

### 2.1. Study design

This study was planned as a randomized clinical trial. The randomization was performed by a physiotherapist blinded to the study using an internet-based method (randomizer.org). The study population comprised 27 participants who were randomized into 3 groups:

- Dorsiflexor (DF) group
- Plantar flexor (PF) group
- Dorsi-plantar flexor (DPF) group.

### 2.2. Participants

The participants included children aged 5–15 years with unilateral or bilateral SCP. Inclusion criteria were being level I or II according to the Gross Motor Function Classification System (GMFCS) [28], and being able to understand and follow simple instructions. Children who had a condition which limited the ability of exercising, had received botulinum toxin injections in the past 6 months, and had undergone an orthopedic operation in the past year were excluded. All of the children and parents provided written informed consent. The study was approved by the ethical review board of Gazi University with approval date and number 26.11.2018-863. The study was conducted between December 1st, 2018, and May 15th, 2019, at Gazi University, Department of Physiotherapy and Rehabilitation, Ankara, Türkiye. The authors conformed to the ethical guidelines of the 1975 Declaration of Helsinki. The study was registered on clinicaltrials.gov with ID: NCT03901703.

### 2.3. Outcome measures

Each participant was assessed individually to avoid rivalry. It was ensured that the participants learned all of the tests correctly before they were performed to eliminate the learning effect. Time was provided between the assessments to ensure at the children had enough time to rest and reduce the effect of fatigue. The assessments were performed by the same therapist, in the same order at baseline, and following 6-weeks of intervention to avoid inter-rater bias.

The assessment of dynamic muscle length was performed using the Modified Tardieu Scale (MTS). This scale measures the point of resistance to a rapid velocity stretch and is performed twice; rapidly and slowly. A ‘catch’ resulting from the ‘overactive stretch reflex’ may be felt in the rapid range of motion (ROM) at a particular angle. This angle was defined as R1. The slow passive joint ROM was measured with goniometry and recorded in a standardized format. This gave an indication of muscle length at rest, or R2. Muscle testing for R1 and R2 was performed using the protocol and test positions stated by Tardieu et al. [29, 30]. A large difference between R1 and R2 implies that there is a large dynamic component due to spasticity, whereas a small difference between them means that there is predominantly fixed contracture in the muscle.

The Pediatric Balance Scale (PBS), a modification of the Berg Balance Scale, was developed as a balance measure for participants [31]. The scale consists of 14 items that are scored from 0 points (lowest function) to 4 points (highest function) with a maximum score of 56 points [32].

The Functional Reach Test was used to measure dynamic balance. The distance that an individual is able to reach forward from a starting standing position with a fixed base of support without loss of balance is measured using a measuring tape at the level of the acromion [33]. This test has shown validity and reliability in children with CP [34].

The Timed Up and Go Test (TUG) measures the time taken by an individual to stand up from a chair, walk a distance of 3 m, turn around, walk back to the chair, and sit down. This test is used in children with CP who are ambulatory to assess dynamic balance and functional mobility [35]. The child was seated with their feet flat on the floor with their hip and knees in 90° flexion. A marking tape was used to stick a mark on the floor at a distance of 3 m from the chair to indicate where the child must turn around. The timing of the test began upon movement to stand after the cue “ready, go” and concluded when the child sat down on the chair. The time needed to complete the test was recorded. The children performed 1 practice trial, followed by 3 test trials. The minimum time was recorded for each trial [36].

For the measurement of sEMG, an 8-channel noninvasive sEMG Noraxon MiniDTS (Noraxon USA Inc., Scottsdale, AZ) system was used. Disposable, self-adhesive Ag/AgCl electrodes (Noraxon Dual EMG Electrode) were used to record the EMG signals. Each electrode was placed parallel to the muscle fibers evaluated, according to the guidelines recommended by the sEMG for the noninvasive assessments of muscles (SENIAM) [37]. The MVCs of the tibialis anterior, gastrocnemius (medial and lateral heads), vastus medialis, and medial hamstrings were measured. The MVCs were recorded by enabling the child to maximally contract the muscle against resistance provided by the therapist. This was done to ensure that the muscle maintained maximum contraction while recording. All of the measurements were performed in 2 trials for each region and the average of these measurements was taken. The raw sEMG signals were passed through 20 Hz, infinite impulse response (IIR), Butterworth high-pass and 500 Hz, IIR, Butterworth low-pass motion artefact filters. Then, the root mean square (RMS) values were calculated from the raw sEMG data in successive time windows (0.1 s) in order to evaluate the signals.

### 2.4. Intervention

The PFE protocol was applied for 6 weeks [21]. Each week, 4 sessions were applied: 2 sessions, at 45 min/session with a physiotherapist, and also 2 sessions/week, for 30 min at home under the supervision of a parent/caregiver. All of the exercises were applied with 3 sets and 10 repetitions in each set. Continuity was checked and necessary adjustments were made by interviewing the parents of the participants weekly.

All of the children, regardless of their group, participated in a functional exercise program consisting of exercises targeting the abdominal and back muscles, hip extensors, abductors, and the quadriceps muscles. The loading of the exercises was increased by altering the ROM of the joints, the difficulty of the exercise, or by changing the height/stability of the surfaces. The description, progression, and photographs of the exercise protocol can be seen in Appendix 1.

#### 2.4.1. DF group exercises

In this group, the participants were given 2 exercises. The first was walking up on a ramp and then walking down on the ramp backwards. The second was dorsiflexion of the feet while sitting with the knees in increasing range of flexion. The difficulty of movement was adjusted by changing the degree of flexion in the knee while sitting.

#### 2.4.2. PF group exercises

Participants in the PF group were given calf raises to train the PF muscles. The difficulty of the exercises was increased by changing the angle of plantar flexion. At the beginning, the movement was initiated from the position where the PF muscles were in their shortened position. At the end of the week 2, the exercise had proceeded to the position where the foot was flat and in contact with the ground. At the end of week 4, plantar flexion was initiated from the position where the muscles were elongated, and the foot was in DF.

#### 2.4.3. DPF group exercises

The participants in this group performed the exercises given in both the DF and PF groups.

### 2.5. Statistical analysis

Statistical analysis was performed using IBM SPSS Statistics for Windows 21.0 (IBM Corp., Armonk, NY, USA). The variables were investigated using visual (histograms, probability plots) and analytical (Shapiro-Wilk test) methods to determine whether they were normally distributed. Nonnormally distributed data were expressed as the median and interquartile range (IQR). The Wilcoxon test was used to compare the changes in the groups. When the level of significance was different in all 3 groups, pre–post measurement differences were determined using the Kruskal-Wallis test. When the level of significance was different in 2 groups, pre–post measurement differences were determined using the Mann-Whitney U test. The level of statistical type-1 error was set as p < 0.05. Based on the results of a previous study, it was estimated that a sample size of 27 participants (9 per group) would have a power of 80% to detect statistically difference in balance analysis (the PBS) for a value of 0.05 [38].

## 3. Results

**Figure 1:**
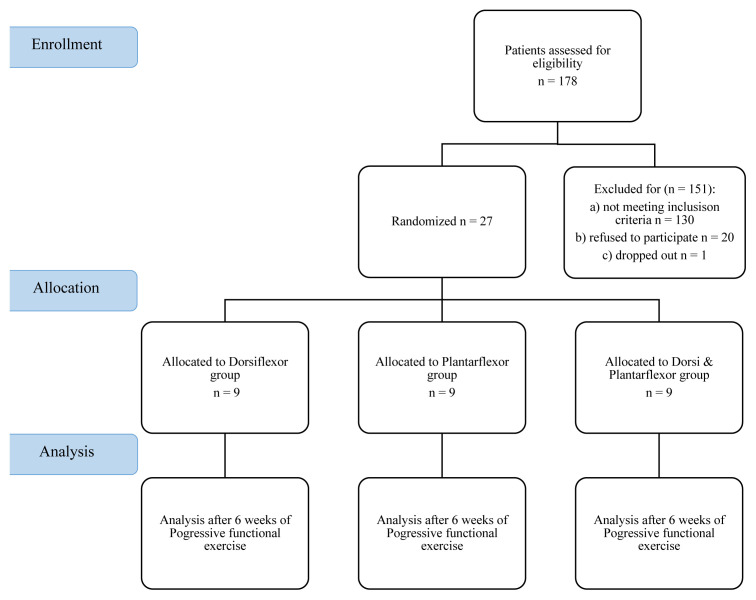
Flow chart of the participation in the study.

A total of 27 participants completed this study, with 9 participants in each group. The flow chart of participation in the study can be seen in Figure 1. Demographic information regarding the participants is shown in Table 1. The participants were included in the rehabilitation program for 4 sessions a week, for 6 weeks. A minimum of 22 and a maximum of 24 rehabilitation sessions were performed per participant. No adverse effect or event was seen in any of the groups. None of the participants reported discomfort or muscle soreness.

When the MTS results were examined, statistically significant differences were observed in the rapid (R1) assessments of the affected side PFs in all of the exercise groups, and in the less affected side PFs of the DF group. Furthermore, it can be seen that there was statistically significant difference in the slow (R2) assessment of the affected side PFs in the DF group. These results can be seen in Table 2, (p < 0.05). When the level of effectiveness between the groups was examined, all three groups were shown to be equally effective on muscle tone (Table 2, p > 0.05).

**Figure 2:**
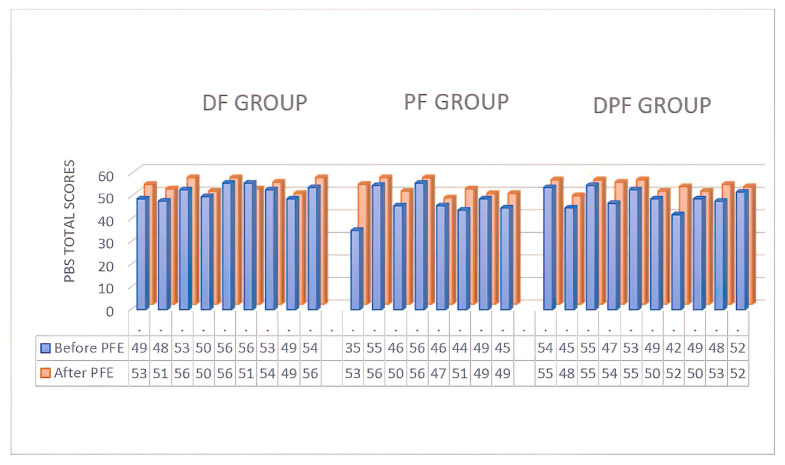
MCID of the PBS results. PBS: Pediatric Balance Scale.

When the PBS results were examined no statistically significant difference was observed in the DF group (Table 3, p > 0.05), however, statistically significant differences were seen in the PF and DPF groups. When the level of effectiveness between PF and DPF groups was examined, it can be seen that there was no difference between the groups, as seen in Table 3, p < 0.05. According to our results, none of the children in the DF group developed at a level of clinical significance (minimal clinically important difference (MCID): 5.83 points) whilst two children in each of the PF and DF + PF groups, had shown development in balance at a clinically significant level. The MCID can be seen in Figure 2. When the functional reach tests were examined, there were statistically significant improvements only in the PF group as seen in Table 3, p < 0.05.

When the TUG test scores were compared, statistically significant changes were found in the DF and PF groups as seen in Table 3 (p < 0.05), however no statistically significant improvement was found in the DPF group (Table 3. p > 0.05). These differences appeared to be equally significant between the groups (Table 3. p > 0.05).

As a result of the sEMG analysis performed to evaluate MVC before and after treatment, significant changes were found in the affected Tibialis Anterior muscle and the less affected Gastrocnemius Medialis muscle in the PF group as seen in Table 4 (p < 0.05). No statistically significant difference was found in the results of the other groups.

## 5. Discussion

This study is the first study to investigate the effects of PFEs of the DF, PF and DPF muscles and interpret the effects of PFEs on these muscle groups separately.

The MTS assessments showed an increase in ROM (R2) in the DF group and an increase in the angle of muscle reaction (R1) in all of the groups. Accordingly, this indicated that there was a significant decrease in spasticity of the PF muscles in all of the groups. Achieving significance in all of the groups revealed that exercises targeting the ankle muscles may generally decrease spasticity. It is our belief that the DF exercises included in this study may have generated an active stretching effect on the PF muscles with every movement, resulting in an increase in the ankle DF joint ROM. The study conducted by dos Santos et al. on closed kinetic chain exercises showed that PFE effectively increase lower extremity muscle strength, thereby facilitating lower extremity contraction and allowing agonist and antagonist muscles to work effectively. As a result, they stated that this led to reduction of muscle tone in the lower extremities [39]. The exercises in the current study are believed to have generated similar results.

This study was the first to investigate the effects of PFEs on balance. It was seen that in groups including plantar flexion exercises, namely the PF and DPF groups, significant changes in the total score of the PBS were evident. When the functional reach test scores were examined, it was seen that statistically significant differences were only established in the PF group. It can be concluded that when PFEs are performed, greater improvements can be achieved in balance. PFEs alone resulted in improvement in static balance. The fact that there was no change in balance as a result of PFEs on the DF group muscles suggests that DF muscles are not as active as PF muscles in balance strategies. Considering that the PF soleus muscle is an important antigravity muscle, the results found herein support these findings [40].

According to the TUG results, there was a significant increase in the functional mobility of the DF and PF groups following the PFEs. The reason for the increase seen in the DF group can be attributed to the development of a more effective gait with the improvement in the DF ROM, and the improvement in the PF group may have been due to the increased balance skills in the PF muscles. No improvement was noted in the DPF group. This finding shows that muscle-specific PFEs lead to better results and that it is necessary to focus directly on a muscle group in order to improve functional mobility. In a study by Schranz et al., 20 patients, aged 6–16 years, with unilateral and bilateral spastic CP were randomly assigned to either progressive resistance exercise or functional exercise groups [41]. According to the results of their study, the increase in strength reached statistical significance only in the functional exercise group. The exercises used in their study were similar to those used in the PF group herein. In addition to their study, significant improvements in balance and TUG were achieved in the current study. In our opinion, this further gain was due to the fact that focused was placed on the foot-ankle area, which is the most affected area in CP patients [42]. From this point of view, the improvements obtained in balance and tone of the PF muscles will contribute greatly to functional abilities.

Lorentzen et al. investigated the effectiveness of gait training performed on an inclined treadmill for 6 weeks in a study involving 32 adults with CP [43]. While the control group continued their routine exercises, the study group practiced walking on an inclined treadmill. As a result of the study, compared to the control group, it was found that there was a statistically significant improvement in gait speed and passive ankle joint stiffness in the experimental group. Walking on an inclined surface was one of the exercises given in the present study, targeting the DF muscles. In addition to these results, statistical improvements were also found in the TUG and muscle tone in the current study. This difference may have occurred due to the younger age of the participants. It is known that, as individuals with CP get older, the severity of the pathologies that occur in their joints increases, their soft tissues adapt to these changes over time and are less sensitive to the interventions. For this reason, it is our belief that an exercise such as walking upwards on a ramp or an inclined surface may have a positive effect on DF muscles when performed at a younger age since the joints may be more open to development with intervention, and this may have led to improvements in the functional tests. In the study by Hye-Jin Cho, 25 participants with CP were randomized into 2 groups, as a functionally progressive resistance exercise group and a control group [27]. The participants in the intervention group participated in functional progressive resistance exercises for 30 min/day, 3 times/week, for 6 weeks. At the end of their study, the intervention group showed improvements in muscle tone, dynamic balance, and functional ability. Thus, when the results of their study and those herein are considered, it can be seen that even 6 weeks of functional training can be beneficial in many aspects. In a study conducted by Ryan et al., children with SCP were included in resistance training of the ankle PFs for 10 weeks. As a result, the authors stated that resistance training did not improve muscle strength, activity, or participation and that adverse effects were seen in 90% of the participants [44]. In the present study, the ankle PFs were trained using functional exercises and body weight only. The loading of the exercises was achieved by changing the foot position and as a result, improvements were seen in balance and functional mobility, a decrease in spasticity was achieved and furthermore, there were no adverse effects in any of the participants. A study by Surana et al. investigated the effects of functional lower extremity exercises in SCP and similar to the current study, they put forth promising results in gait capacity and performance [45]. Current guidelines on exercise interventions in CP also state that exercises must be functional and in a real-life context [46]. Therefore, in our opinion, PFEs may be more beneficial than resistance exercises in the ankle area.

In the current research, the MVC results showed that the muscles included in the sEMG exhibited different degrees of activity. While there were increases in the MVCs of some of the muscles, decreases were evident in others. In our opinion, the significant changes found in the affected tibialis anterior muscle and the less affected gastrocnemius medialis muscle in the PF group occurred coincidentally, and an interpretation is not possible due to many factors. In the literature, studies using sEMG have reported that reciprocal inhibition is insufficient in patients with CP and may cause problems during muscle relaxation, leading to errors in MVC. Furthermore, it was reported that obtaining isolated muscle contraction in patients with SCP is very difficult [47]. According to previous sEMG studies, there may be coactivations in the muscles, which may also lead to errors in the results [48, 49]. Rose et al. stated that type I muscle fibers are more common in the muscles of patients with SCP. High muscle fiber firing cannot be achieved in MVC measurements, and therefore, may also lead to errors in measurements [49]. In addition to these factors, we believe that the pathological changes occurring in the muscles in patients with SCP may have caused a shift in the reference points, which are important for accuracy in sEMG measurements. Superficial electrodes are placed on the motor points of the relevant muscles. It was reported by SENIAM that the most accurate measurements can be made from these points [37]. However, these reference points are determined for healthy individuals who show typical development. It is known that pathological changes that directly affect muscle fibers in SCP lead to elongation in the sarcomere length of the muscles and changes in the fiber type [50]. Furthermore, it was reported that motor points in the muscles of patients with CP may be at different points in the muscle compared to their healthy peers [51]. These factors may have led to an increase in the signals received from neighboring regions during measurements, which is known as *physiological crosstalk*. It is our belief that there may not have been a significant difference in muscle activation due to these factors.

Even though the sEMG results in the current study showed that there was no statistically interpretable difference in the MVC, it can be seen that PFEs led to positive effects on function, muscle tone, and balance according to the muscle group focused on. Boyd et al. reported that in order to achieve functional development in patients with CP, exercises must consist of functional movements and should be practiced in a context-specific manner [46]. The present research supports these findings. Therefore, we believe that this study will guide physiotherapists working in the rehabilitation of patients with CP on how to achieve beneficial results with functional exercise.

This study had some limitations. Although there was an improvement in function, the effect of the PFE on strength was not measured. Such effects could have been reported objectively with the use of a reliable method to measure muscle strength. Furthermore, a larger sample size may have yielded more power in detecting statistically significant relationships. In future studies, the effectiveness of PFEs on strength should be investigated with a longer exercise duration, with objective measures for strength, and a larger sample size.

The results herein revealed that functional improvement is exercise-specific. Gains are obtained according to the function of the muscle group. It was seen that the PFEs of the PF muscles of patients with CP led to an improvement in balance, whereas the PFEs of the DF muscles led to an increase in the ROM of the ankle joint. When PFEs were performed on both the DF and PF muscles, the effects of both exercise groups appeared in combination; however, the results were not as effective as exercising the PF muscles individually.

## Figures and Tables

**Table 1:** Demographic characteristics and functional status of the participants.

		DF group median (IQR) n: 9	PF group median (IQR) n: 9	DPF group median (IQR) n: 9	p-value
Age (years)		8 (6.5/12.5)	8 (6.5/13)	6 (5.5/11.5)	0.599
Height (cm)		130 (115.5/156.5)	136 (120/151)	120 (116.5/154.5)	0.759
Weight (kg)		23 (18.5/49)	28 (23.5/33)	20.8 (18.3/55.4)	0.832
		**n (%)**	**n (%)**	**n (%)**	
Sex	Male	5 (55.6)	4 (44.4)	6 (66.7)	0.648
	Female	4 (44.4)	5 (55.6)	3 (33.3)	
GMFCS	I	6 (66.7)	4 (44.4)	4 (44.4)	0.565
	II	3 (33.3)	5 (55.6)	5 (55.6)	

DF: Dorsiflexor, PF: plantarflexor, DPF: dorsi-plantar flexor, IQR: inter quartile range, cm: centimeters, kg: kilograms, GMFCS: Gross Motor Function Classification System.

**Table 2:** Comparison of the MTS measurements within and between the groups before and after PFE treatment.

		DF group n: 9				PF group n: 9				DPF group n: 9				
Plantarflexors		Before median (IQR)	After median (IQR)	p-value within groups	Change (Δ) median IQR	Before median (IQR)	After median (IQR)	p-value within groups	Change (Δ) median IQR	Before median (IQR)	After median (IQR)	p-value within groups	Change (Δ) median IQR	p-value between groups
Affected side	Slow (R2) (°)	10 (3.5/12.5)	15 (10/20)	*0.015		10 (7/15)	10 (10/20)	0.197		10 (2.5/20)	15 (9/20)	0.104		
	Rapid (R1) (°)	0 (−1.5/7.5)	12 (7.5/17.5)	*0.008	5 (0/6.5)	5 (1/9.5)	10 (5/15)	*0.042	5 (0/15)	0 (−7.5/7.5)	10 (9/15)	*0.017	0 (0/0)	0.140
	Difference	5 (0/10)	0 (0/6.5)	0.092		6 (3/9.5)	5 (0/7.5)	0.260		10 (5/15.5)	5 (0/7.5)	*0.049		
Less affected side	Slow (R2) (°)	20 (11.5/20)	20 (15/20)	0.465		15 (10/20)	20 (13.5/20)	0.465		20 (17.5/20)	20 (10/20)	0.102		
	Rapid (R1) (°)	10 (10/20)	20 (15/20)	*0.039		10 (5/11)	15 (11/20)	0.092		20 (15/20)	20 (10/20)	1.000		
	Difference	0 (0/7.5)	0 (0/0)	0.066		5 (0/10)	0 (0/0)	0.206		0 (0/2.5)	0 (0/0)	0.180		

(°): Degrees, R1: the first point of resistance felt by the therapist due to the catch resulting from the overactive stretch reflex; R2: the passive joint ROM.

* Indicates that there was a statistically significant difference (*p < 0.05 within the groups after 6 weeks of PFE treatment [Wilcoxon signed-rank test] and changes between the groups [Kruskal-Wallis test]).

**Table 3:** Comparison of the balance and functional test measurements within and between the groups before and after PFE treatment.

Before median (IQR)		DF group n: 9				PF group n: 9				DPF group n: 9			
		After median (IQR)	p-value within groups	Change (Δ) median IQR	Before median (IQR)	After median (IQR)	p-value within groups	Change (Δ) median IQR	Before median (IQR)	After median (IQR)	p-value within groups	Change (Δ) median IQR	p-value between groups
Lateral reach (cm)	Affected side	28 (23.5/29.5)	29 (24.5/32.5)	0.307		22 (12/28)	29 (23/31)	*0.008		24 (15.5/28)	19 (16.5/32)	0.398	
	Less affected side	29 (23.5/33)	30 (26.5/34.5)	0.173		23 (13/30)	30 (25/31)	*0.011		27 (17/35.5)	19.5 (25/33)	0.888	
PBS		53 (49/55)	53 (50.5/56)	0.344		46 (44.5/54.5)	51 (49/56)	*0.017	1 (0.5/5.5)	49 (46/52.5)	52 (50/5405)	*0.018	2 (0.5/6)
TUG		6.68 (5.28/7.85)	5.68 (5.01/6.50)	*0.038	−0.68 (−1.56/−0.08)	7.45 (5.76/8.65)	6.62 (5.53/7.83)	*0.042	−0.23 (−0.74/0)	6.16 (6/8.06)	6.22 (5.60/7.13)	0.660	

PBS: Pediatric Balance Scale, TUG: Time Up and Go test.

* Indicates that there was a statistically significant difference (*p < 0.05 within the groups after 6 weeks of PFE treatment [Wilcoxon signed-rank test] and changes between the groups [Mann-Whitney U test]).

**Table 4:** Comparison of the MVC of the muscle measurements with sEMG within the groups before and after PFE treatment.

		DF group n: 9			PF group n: 9			DPF group n: 9		
		Before median (IQR)	After median (IQR)	p-value within groups	Before median (IQR)	After median (IQR)	p-value within groups	Before median (IQR)	After median (IQR)	p-value within groups
Vastus medialis obliquus	Affected side	9.2 (3.5/46.5)	28.5 (7.1/57.8)	0.374	49.4 (16.3/110.3)	13.9 (6.1/47.8)	0.314	57.2 (42.9/74.4)	39.8 (20.4/61.8)	0.214
	Less affected side	46.7 (13.7/61.3)	27.3 (6.5/52.1)	0.173	71.9 (4.5/106.2)	10.4 (3.5/81.1)	0.038	46.8 (7.9/61.8)	41.6 (12.1/64.2)	0.889
Semitendinosus	Affected side	21.9 (9.9/87)	34 (12.1/71.8)	0.859	10.9 (6.4/98.2)	7.3 (5/28.8)	0.260	38.3 (21.3/80)	35.9 (13.4/87.9)	0.515
	Less affected side	17.6 (11.7/71)	28.8 (12.9/76.7)	0.953	60.6 (5.9/144.5)	12.5 (5.4/68.6)	0.173	55.9 (16/144.3)	59 (37.4/106.3)	0.767
Tibialis anterior	Affected side	53.6 (14.8/123.8)	15 (9.7/30)	0.051	66.8 (49.3/85.7)	30.5 (5.2/47)	*0.021	26.4 (10.1/87.8)	40.4 (13.2/59.3)	0.374
	Less affected side	83.6 (15.7/156)	49.9 (11.7/160.5)	0.678	66.8 (34.9/162)	37.8 (20/77.9)	0.051	78.2 (13.6/132.5)	92.7 (12.6/145.3)	0.678
Gastrocnemius medialis	Affected side	17.3 (8.2/40.3)	16.8 (6.6/46.9)	0.859	17.6 (4.3/73)	13.5 (4.1/48.8)	0.594	10.1 (3.8/38.7)	6.8 (2.2/29.4)	0.173
	Less affected side	15.9 (3.8/32.9)	40.8 (8.2/65.2)	0.173	79.6 (20.8/105)	25.2 (9.8/39.1)	*0.021	15 (6.5/73.5)	21.3 (5.8/71.7)	0.767
Gastrocnemius lateralis	Affected side	14.4 (5.1/33.7)	30.3 (10.5/43.1)	0.374	7.9 (3.2/47.3)	10 (4.7/33.3)	0.767	16.8 (6.9/25.1)	22.7 (4.4/41.3)	0.374
	Less affected side	26.7 (7.4/65.2)	36.7 (18.7/53)	0.441	51.9 (10.2/103.7)	40.9 (8.9/56.9)	0.110	27.1 (5.8/36)	14.1 (6.2/28)	0.441

* Indicates that there was a statistically significant difference (*p < 0.05 within the groups after 6 weeks of PFE treatment [Wilcoxon signed-rank test]).

## Appendix The Progressive functional exercise protocol

### 1. Stepping up on a 20 cm step

[Fig f3-turkjmedsci-53-5-1166]

### 1. Stepping up sideways on a 20 cm step

[Fig f4-turkjmedsci-53-5-1166]
